# Supplementary material for: Immunomodulation of Pluripotent Stem Cell-Derived Mesenchymal Stem Cells in Rotator Cuff Tears Model
Source: Biomedicines. 2022 Jun 29;10(7):1549. doi: 10.3390/biomedicines10071549 (PMC9312476; doi:10.3390/biomedicines10071549)
Supplement: Supplementary file 1 [file biomedicines-10-01549-s001.zip › biomedicines-1653461-supplementary.pdf]

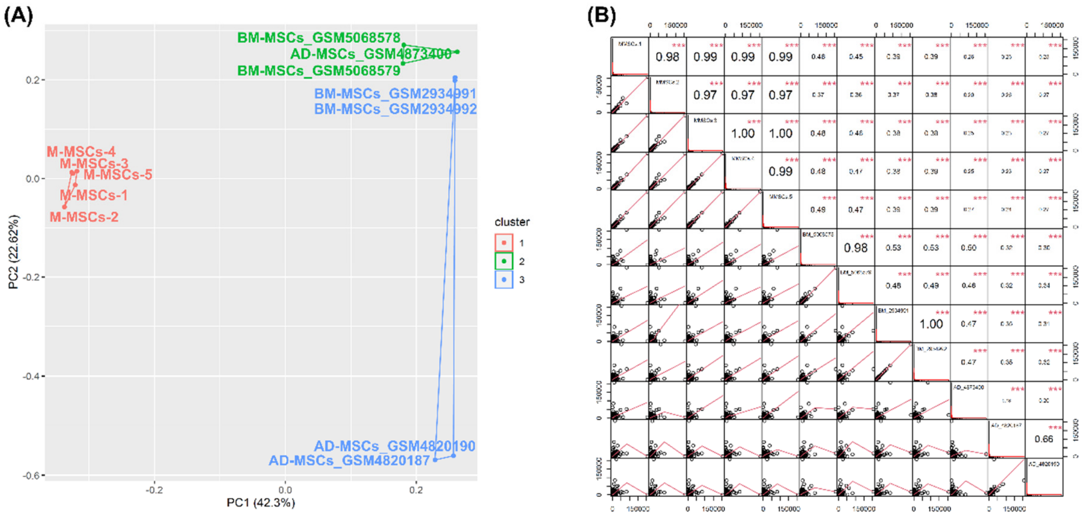

Supplementary Figure S1. Transcriptomic analysis of M-MSCs compared to adult MSCs. (A) Principal component analysis (PCA) with clustering for M-MSCs, BM-MSCs, and AD-MSCs. Relative distances of the cells visualized as dots with colors. Each dot represents each model. Clustered samples share the colors and lines. (B) Correlogram with the significance values between M-MSCs, BM-MSCs, and AD-MSCs. The gene expressions of the cells are scattered as dots and their distribution is shown on the diagonal. Bivariate scatter plots with a fitted line are displayed on the bottom of the diagonal, and the values of the correlation and their significance level are displayed on the top of the diagonal ($p < 0.01$, ***). The BM-MSCs and AD-MSCs are presented with their NCBI GEO GSM numbers.

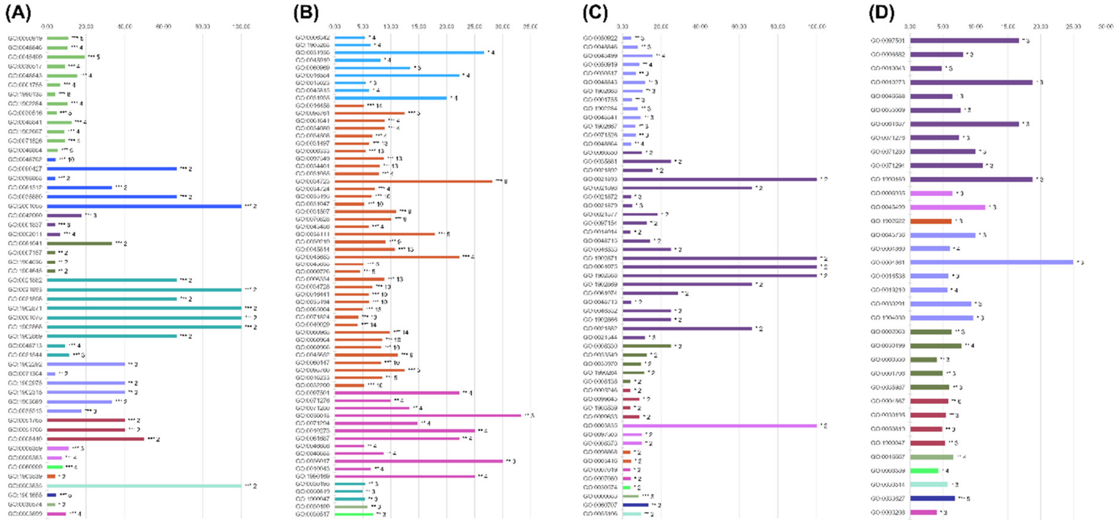

Supplementary Figure S2. The percentages of genes per GOs for each DEG. (A) Upregulated DEGs in M-MSCs compared to BM-MSCs. The fold changes (FCs) were over 4. (B) Downregulated DEGs in M-MSCs compared to BM-MSCs. The FCs were under 0.25. (C) Upregulated DEGs in M-MSCs compared to AD-MSCs. The FCs were over 4. (D) Downregulated DEGs in M-MSCs compared to AD-MSCs. The FCs were under 0.25 (*, $p < 0.05$; **, $p < 0.01$; ***, $p < 0.001$).

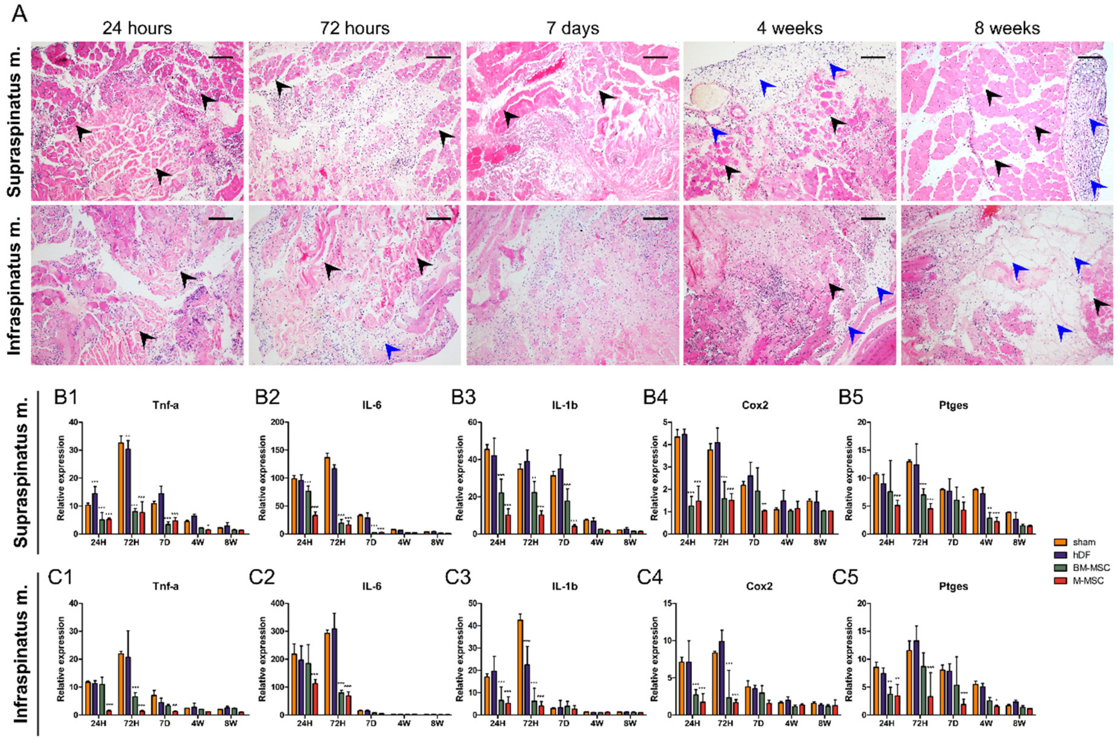

Supplementary Figure S3. Morphological evaluation and relative expressions of genes related to inflammation within the sham group

Muscle samples were excised from hDFs, BM-MSCs, sham and M-MSCs groups at 24 h, 72 h, 7 days, 4 weeks, and 8 weeks after RCT induction. (A) Supraspinatus and infraspinatus muscles were stained with hematoxylin and eosin. Inflammatory lesions are observed with severe mononuclear cell infiltration. Black arrows indicate atrophy, and blue arrows indicate fat infiltrates. The scale bars are 100 μm. (B-C) Genes related to inflammation include (B1, C1) TNF-a, (B2, C2) IL-6, (B3, C3) IL-1b, (B4, C4) Cox2 and (B5, C5) Ptges were monitored. The data are shown as mean ± SE and the significance was represented as *, $p < 0.05$; **, $p < 0.01$; ***, $p < 0.001$, respectively.

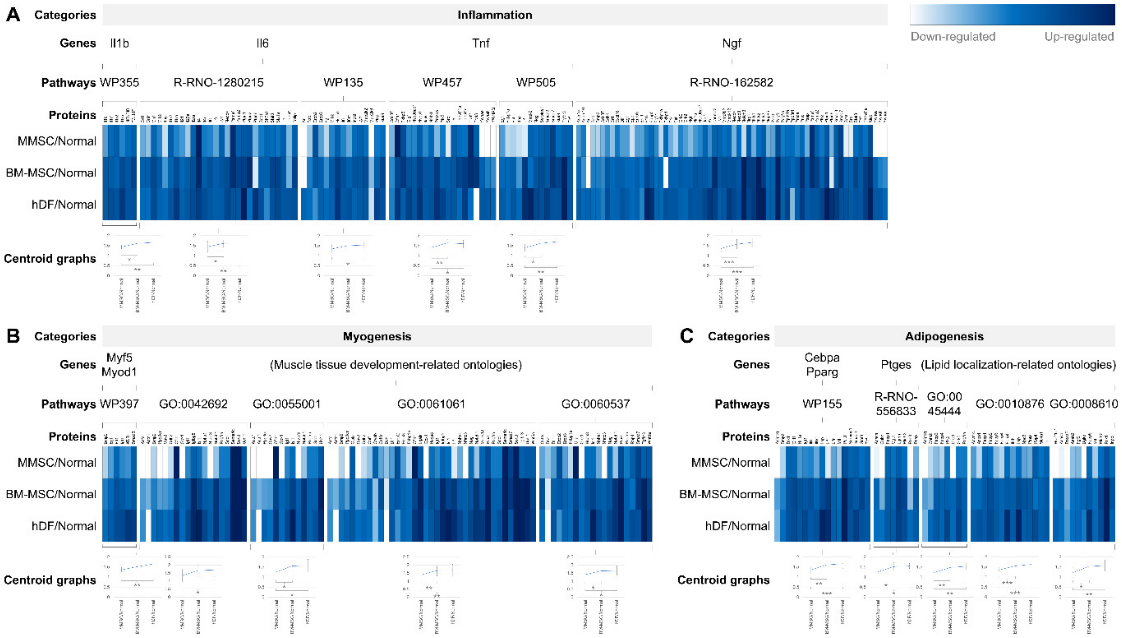

Supplementary Figure S4. Antibody-Based Protein Microarray

The slide scanning was performed using GenePix 4100A Scanner (Axon Instrument Inc., Foster City, CA, USA). The slides were absolutely dried before the scanning and scanned within 24-48 hours. The slides were scanned at 10 μm resolution, optimal laser power, and PMT. After getting the scanned image, they were grided and quantified with GenePix Software (Axon Instrument Inc., Foster City, CA, USA). After analyzing, the data about protein information was annotated using UniProt DB and were quantile normalized. (A, B, C) The heatmaps were visualized with fold changes of MMSC-treated RCT muscle/normal muscle, BM-MSC-treated RCT muscle/normal muscle, and hDF-treated RCT muscle/normal muscle for ontologies/pathways related to inflammation, myogenesis, and adipogenesis. The ontology/pathway terms were searched from EMBL-EBI [32], WikiPathways [13], and Reactome[33].

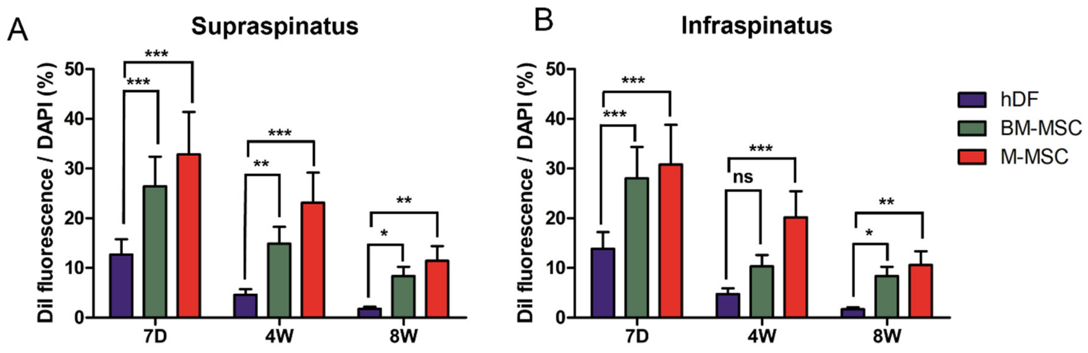

Supplementary Figure S5. Comparison of long-term in vivo MSC tracking based on DiI. Supraspinatus and infraspinatus muscles were examined from hDF (control), BM-MSC and M-MSC groups at 7 days, 4 weeks, and 8 weeks after RCT induction. The expression level of DiI+ compared to the amount of DAPI expression was expressed as a percentage. (A) is a graph showing the amount of DiI+ expression compared to the DAPI expression level in supraspinatus as a percentage, (B) is a graph expressing the DiI+ expression level compared to the DAPI expression level in infraspinatus as a percentage. The data are shown as mean ± SE and the significance was represented as *, $p < 0.05$; **, $p < 0.01$; ***, $p < 0.001$, respectively.

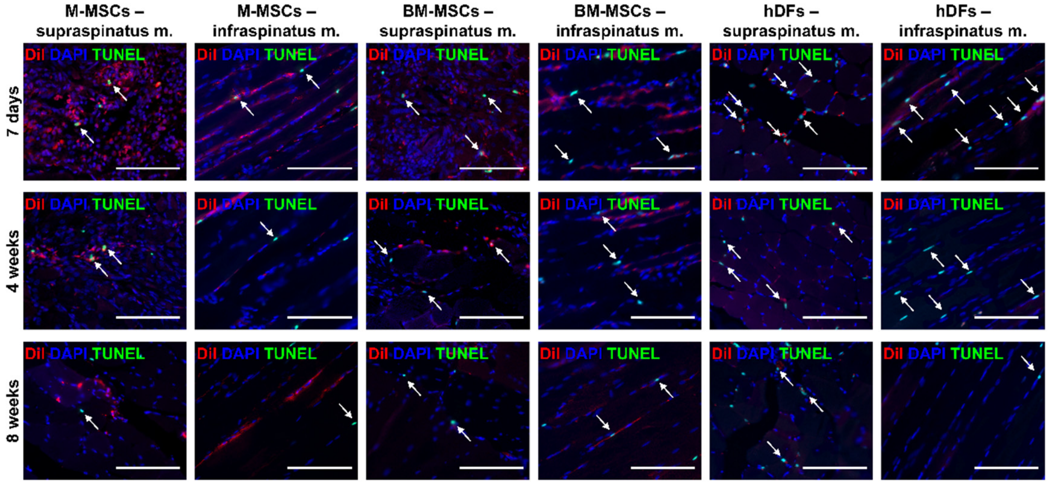

Supplementary Figure S6. Comparison of apoptosis in treated cells by TUNEL staining

Supraspinatus and infraspinatus muscles were examined from hDF (control), BM-MSC and M-MSC groups at 7 days, 4 weeks, and 8 weeks after RCT induction. All nuclei were determined in DAPI blue, DiI+ cells in red, and TUNEL+ cells in green. Arrows indicate DiI+, TUNEL+ cells. The scale bars are 100 μm.
